# Surface Roughness Evaluation Based on Acoustic Emission Signals in Robot Assisted Polishing

**DOI:** 10.3390/s141121514

**Published:** 2014-11-14

**Authors:** Beatriz de Agustina, Marta María Marín, Roberto Teti, Eva María Rubio

**Affiliations:** 1 Department of Manufacturing Engineering, Industrial Engineering School, National University of Distance Education (UNED), C/Juan del Rosal, 12, E28040-Madrid, Spain; E-Mails: mmarin@ind.uned.es (M.M.M.); erubio@ind.uned.es (E.M.R.); 2 Department of Materials and Production Engineering, University of Naples Federico II Piazzale Tecchio, 80, Naples 80125, Italy; E-Mail: roberto.teti@unina.it

**Keywords:** robot assisted polishing, surface roughness, acoustic emission signals, contact force, monitoring

## Abstract

The polishing process is the most common technology used in applications where a high level of surface quality is demanded. The automation of polishing processes is especially difficult due to the high level of skill and dexterity that is required. Much of this difficulty arises because of the lack of reliable data on the effect of the polishing parameters on the resulting surface roughness. An experimental study was developed to evaluate the surface roughness obtained during Robot Assisted Polishing processes by the analysis of acoustic emission signals in the frequency domain. The aim is to find out a trend of a feature or features calculated from the acoustic emission signals detected along the process. Such an evaluation was made with the objective of collecting valuable information for the establishment of the end point detection of polishing process. As a main conclusion, it can be affirmed that acoustic emission (AE) signals can be considered useful to monitor the polishing process state.

## 1. Introduction

The increasing demand for improved surface integrity of manufactured components with high production rate has acted as a driving force in the development of automated production methods. For this reason, the monitoring of surface quality obtained by a manufacturing process has been extensively studied and improved in the last years. In fact, a great effort has been made to evaluate and control on line the surface roughness obtained in finishing machining processes by the analysis of signals detected by different sensors such as dynamometer, acoustic emission, accelerometer, current/power among others [1–7]. This is an important challenge to avoid valuable product rejects and refinishing [8,9].

Among these processes, polishing is the most common technology used in those applications in which high surface qualities, in terms of low roughness level, minimized subsurface damage and high form accuracies, are demanded. It is an essential step in optics manufacturing and in mold finishing operations [4].

The automation of polishing is especially difficult due to the high level of skill and dexterity that is required. Much of this difficulty arises due to a lack of data in relation with the effect of the polishing parameters on the resulting surface roughness, although some experimental works have been published in this area [10,11].

In general, these studies built up a specific database with experimental data and working knowledge that includes a set of polishing conditions such a tool type, size and grit, feed rate, number of passes and cutting fluids used [12–14].

Until now, regarding the monitoring of polishing processes, there are few experimental studies carried out [12,15–21].

In some of them, sensors have been integrated during the automated polishing processes. Among them, the use of acoustic emission sensors has allowed the achievement of valuable results for the estimation of the process state [12,17–21].

In this work, an experimental study was developed to evaluate the surface roughness obtained during robot assisted polishing processes by the analysis of acoustic emission (AE) signals. The aim is to find out a trend of a feature or features calculated from the AE signals obtained along the process. Such an evaluation was made with the objective of collecting valuable information for the establishment of the end point detection of polishing process; this is when the required surface roughness is reached or it is necessary to replace the tool and select a finer one and/or change other polishing condition, to carry on with the following stage of polishing.

For this propose, dry polishing turning tests were carried out on a Robot Assisted Polishing machine (STRECON NanoRAP 200) performed during five polishing sessions, at different polishing contact force, using the same polishing stone tool. Along the tests, AE signals were acquired and roughness surface measurements were taken at the end of each polishing session.

## 2. Experimental Procedure

The experimental procedure taken in this study is shown on the following points:

### 2.1. Polishing Tests

For the development of the tests, it is proposed to employ a Robot Assisted Polishing (RAP) system. This is a polishing machine equipped with a robot arm in which interchangeable polishing tools are mounted. A control module is integrated in the robot arm that allows the setting of, not only the conventional polishing parameters such a cutting speed and feed rate, also the contact force between the tool and workpiece, and the tool pulsation. This movement increases the cutting efficiency and improves the steadiness of the machining process.

**Figure 1.**
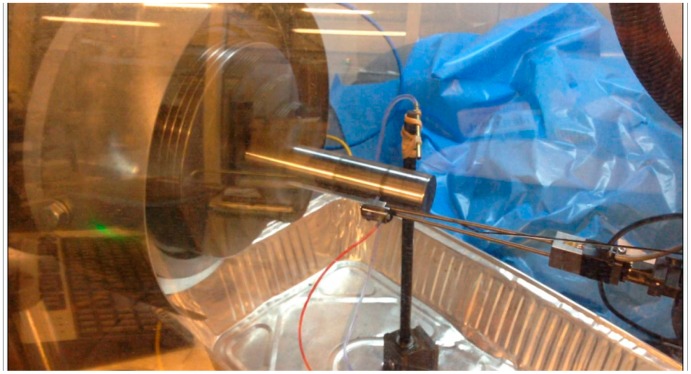
Robot Assisted Polishing machine.

In this study, dry polishing turning tests were carried out on a Robot Assisted Polishing machine (STRECON NanoRAP 200) performed during five polishing sessions, at different polishing contact force. Experimental tests were taken on a bar of alloy steel (UNS G52986) with a length of 75 mm and diameter of 30 mm (Figure 1). This machine operates similarly to a turning lathe. In this case, the operation performed was a cylindrical turning in which three movements are combined: the rotation of the workpiece, the feed movement of the tool and a third movement; the tool has also a pulsation movement on the feed direction with a frequency of 500 pulses/min on a length of 1 mm.

The abrasive tool selected was the polishing stone with a grit number of 800 that corresponds to a grain size of approximately 11 μm from Gesswein (MP800) according to the Comercial Standard CS271-65 [22].

Each session consists of 60 polishing passes. The Table 1 summarizes polishing conditions used in each session.

### 2.2. Acquisition of AE Signal during Polishing Tests

An AE sensor was mounted on the tool holder as close as possible to the polishing stone to minimize signal loss and achieve a good signal-to-noise ratio. Signals samples were taken with an interval time between acquisitions of, approximately, 0.93 s. That is, along the five polishing sessions, a total number of 4650 acquisitions. Sample rate was set up for 1 MHz.

### 2.3. Surface Roughness Measurements

At the end of the each polishing session, five surface roughness measurements were taken on the workpiece (along one of its generatrix) by the use the roughness tester MahrSurf XD1 equipped with a 2 μm radius tip. For the measurements, it was applied a cut-off and an evaluation length of 0.25 and 1.25 mm, respectively.

In the measurement process, data (xi, zi) of the surface geometry workpiece were obtained. The arithmetical average roughness, *Ra*, was selected as a parameter to analyze. According to ISO 4288 (ISO 4288, 1996) [23] standards, this parameter is defined as the arithmetical average of the absolute values of the deviations of the roughness profile, *R*, and it is expressed mathematically by means of the Equation (1):

(1) $$Ra=\frac{1}{l_{m}}\int_{0}^{l_{m}}\left|z(x)\right|dx$$

### 2.4. Analysis of AE Data

Row AE signal data arrays, with 131,072 values each of them, are stored and analyzed by Matlab software. Different features were calculated to determine the process state, in terms of surface roughness, along the polishing time.

From AE data, an analysis in the frequency domain was carried out by using the Fast Fourier Transform (FFT) algorithm. This is an efficient algorithm that allows the representation of a digital signal in the frequency domain using less number of numerical operations compared to Digital Fast Fourier (DFT) with a substantial computational power saving [24].

Firstly, FFT power spectral graphics at different polishing time were plotted. The objective is to select a range or ranges of frequencies where the amplitudes of certain peaks shown in the FFT power spectral plots decrease with the time process, as it is pointed out in previous studies in which robot assisted polishing tests were carried out [18,24]. Once the range of frequencies was selected, maximum amplitude was calculated from all the AE acquisitions registered to achieve a possible trend and a correlation with the surface roughness.

## 3. Results

As a first approach, FFT power spectral plots were obtained at different polishing time corresponding to the acquisitions taken, approximately, at the middle of each polishing session.

**Figure 2.**
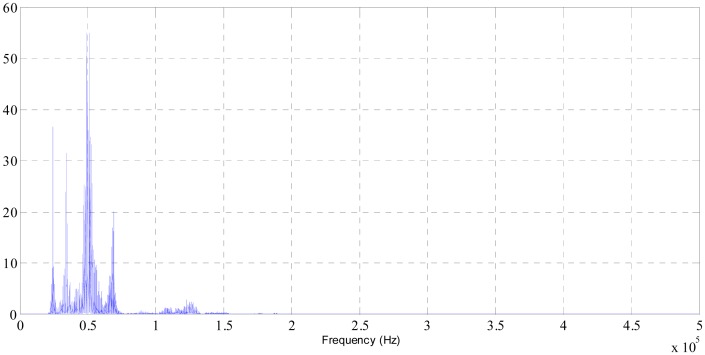
Fast Fourier Transform (FFT) power spectral plot at 0400 acquisition (session 1).

All of them show several peaks that appear around the same range of frequencies as it can be seen in Figure 2. This is the FFT power spectral plot at 0400 acquisition during the session 1. As it was mentioned, the amplitude of some of those peaks decreases as process progresses, whereas magnitudes on other frequencies do not show a variation over time.

In addition, it is important to remark that when a higher magnitude of contact force was applied, the amplitudes of these peaks increased. This direct relationship, between the selected AE signal parameter and the contact force, was observed at the last session of polishing (session 5).

Further analysis was made to evaluate the whole polishing process; this is the total acquisitions taken during the five sessions. According to the peaks shown in the FFT power spectral representations, different ranges of frequencies were selected to determine the maximum amplitude of the different peak reached. Among the ranges of frequencies analyzed (ranges that contain peaks at 24, 34 50, 69 and 125 kHz) the selected range that is shown in this study, is the range between 60,000 and 100,000 Hz, so a clearer trend was found. Similar results are presented in the study developed by Pilný and partners [18], in which three main peaks were distinguished at 20, 45 and 150 kHz.

**Figure 3.**
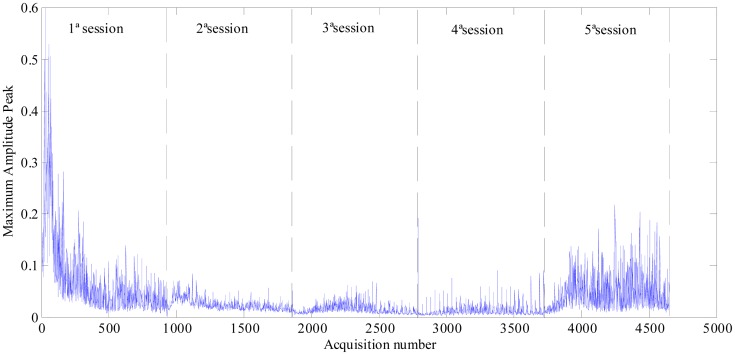
Maximum amplitudes of FFT power spectral of acoustic emission (AE) signal (60,000–100,000 Hz).

Maximum amplitudes calculated from each acquisition along the five polishing sessions are plotted in the following Figure 3.

As it can be observed, as the polishing process progresses, the amplitudes tend to decrease, apart from the ones obtained at the fifth session, for the reason that was previously pointed out.

Besides, at the end of each polishing session, five surface roughness measurements were taken on the workpiece, around the same location.

**Figure 4.**
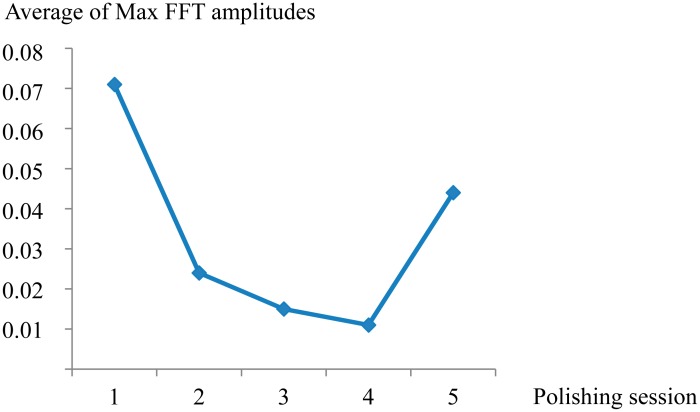
Plot of the average of maximum amplitudes FFT power spectral of AE signal *vs.* polishing session.

**Figure 5.**
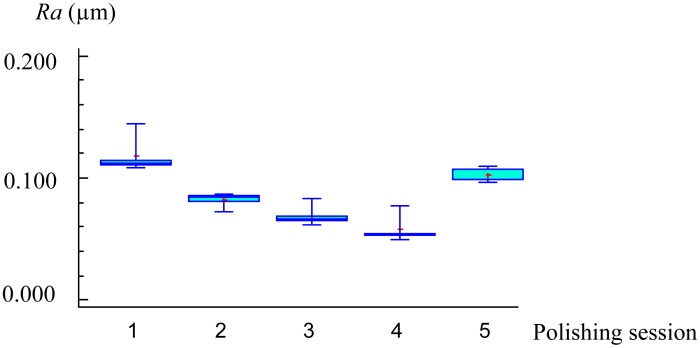
*Ra* measurements at the end of each season.

Finally, to compare the results from the analysis of AE signals in the frequency domain and the obtained surface roughness, the averages of maximum amplitudes of FFT power spectral were plotted in Figure 4 and the five *Ra* measurements for each polishing season were plotted by means of box and whisker diagrams (Figure 5).

First of all, it is important to clarify with respect the Figure 5, that the values of the surface roughness measured on the workpiece before polishing is included and plotted at the polishing session number 0.

As shown in Figures 4 and 5, the feature extracted of AE signals and the surface roughness follow the same trend, obtaining the lowest value of *Ra* (0.059 μm) at the fourth polishing session. This is when it would be approached the end detection of the process. Afterwards, at the fifth polishing session, with the increase of the contact force applied up to 17.64 N, a larger value of *Ra* was obtained. Nevertheless, at first stage of polishing process (session 1) with the use of a higher magnitude of contact force, at the same cutting condition, the surface roughness improved from 0.142 to 0.116 μm. This shows that it is necessary to reduce such control parameters for the last polishing stages.

## 4. Conclusions

As a first approach, in this study, an evaluation of the surface roughness obtained by Robot Assisted Polishing with different contact force applied between the tool and the workpiece, was developed. Concretely, dry polishing turning tests were carried out on a bar of alloy (UNS G52986) by using a tool with a grit number of 800. An analysis of the AE signal in frequency domain detected during the process was made with the objective of finding a feature or features from AE signal sensitive to the variations of the surface roughness measured along five polishing sessions. The main conclusions extracted from the results obtained in this study can be summarized as follows:

‐ For the polishing conditions employed in this study, the surface roughness and the analyzed feature calculated from the frequency domain of AE signal (maximum amplitude of the FFT power spectral between the frequencies 60,000 and 100,000 Hz) during polishing process follow the same trend. Therefore, AE signal can be considered useful for the monitoring of polishing process state.
‐ The end point detection of the process was determined at the end of the polishing session 4. That corresponds to the polishing stage when the most improved surface roughness has been reached.
‐ The contact force between the tool and the workpiece is a control parameter of the process that should be reduced at the last polishing stages.
‐ Finally, it is important to indicate that from the results obtained in this study, it is possible to collect valuable data to use together with other features extracted from others sensors to further develop monitoring polishing systems.

## Figures and Tables

**Table 1.** Polishing conditions at the five sessions performed.

**Polishing Session**	**Contact Force (N)**	**Spindle Speed (rpm)**	**Feed Rate (mm/s)**	**Pulsation Tool (Pulses/min)**	**Pulsation Stroke (mm)**
1	17.64	300	5	500	1
2	9.8				
3	9.8				
4	9.8				
5	17.64				
